# Infective Endocarditis in Septic Shock: Results From an Observational Multicenter Study

**DOI:** 10.7759/cureus.78927

**Published:** 2025-02-13

**Authors:** Saad LNU, Asif Jamal, Muhammad A Raza, Zeeshan Umar, Sohail Ahmad, Wafa Alvi, Lintha Z Khattak, Aamir Ajmal, Naqeeb Ullah, Kainat Khan

**Affiliations:** 1 General Medicine, Lady Reading Hospital Medical Teaching Institution, Peshawar, PAK; 2 Internal Medicine, Bukhari Medical and Surgical Complex, Bannu, PAK; 3 Cardiology, Sardar Fateh Muhammad Khan Buzdar Institute Of Cardiology, Dera Ghazi Khan, PAK; 4 Emergency Medicine, Pak Emirates Military Hospital, Rawalpindi, PAK; 5 Internal Medicine, Lady Reading Hospital, Peshawar, PAK; 6 Emergency Medicine, POF Hospital Wah Cantt, Wah Cantt, PAK; 7 Surgery, Khyber Medical College, Peshawar, PAK; 8 Neurology, Rehman Medical Institute, Peshawar, PAK; 9 Resident Physician, Lady Reading Hospital, Peshawar, PAK

**Keywords:** emergency department, infective endocarditis, multicenter study, outcomes, septic shock

## Abstract

Introduction

Infective endocarditis (IE) in patients with septic shock poses diagnostic challenges due to overlapping systemic effects and comorbidities, making early recognition crucial for improving outcomes. This study aimed to characterize the clinical features, diagnostic findings, and outcomes of IE in septic shock to inform better early recognition and management strategies in emergency and critical care settings.

Methodology

A multicenter observational study was conducted across three tertiary care hospitals in Pakistan over two years, involving 300 patients presenting with septic shock and confirmed IE. Adults aged 18 years or older who met the Sepsis-3 criteria and were diagnosed with IE using the modified Duke criteria were included. Data collected included demographics, clinical characteristics, imaging results, blood cultures, inflammatory markers, treatment plans, and outcomes (e.g., mortality, embolic events, ICU admission). Multivariate analysis identified independent predictors of adverse outcomes, adjusting for confounders such as age and comorbidities. Missing data were addressed using multiple imputations, which allows for the creation of several plausible datasets to account for uncertainty and minimize bias. This method was selected over simpler approaches, such as mean or median imputation, to enhance the robustness of our findings.

Results

The mean age was 55.20 ± 14.70 years, the incidence of echocardiographic positivity was 96.33% (n=289), and blood culture positivity was 92.67% (n=278). At admission, patients exhibited varying degrees of septic shock severity based on hemodynamic instability, with hypotension (83.33%) being a prominent feature. Fever (90%) and dyspnea (60%) were among the most frequently reported symptoms. Diagnostic challenges were encountered in 12% of cases, where initial differential diagnoses excluded IE but were later revised based on echocardiographic and microbiological confirmation. Pathogens predominantly included *Staphylococcus aureus* (50%) and *Streptococcus* species (30%), with polymicrobial infections noted in 8% of cases. Complications included embolic events (33%), heart failure (28%), renal dysfunction (25%), and neurological involvement such as stroke (10%). ICU admission was required in 50% (n=150) of cases, and in-hospital mortality occurred in 17% (n=51). Predictors of adverse outcomes included older age (AOR: 1.05, 95% CI: 1.02-1.08, p=0.001), prior cardiovascular disease (AOR: 2.14, 95% CI: 1.12-4.08, p=0.021), echocardiographic positivity (AOR: 2.43, 95% CI: 1.36-4.34, p<0.001), blood culture positivity (AOR: 2.50, 95% CI: 1.43-4.34, p<0.001), embolic events (AOR: 3.10, 95% CI: 1.86-5.14, p<0.001), and elevated inflammatory markers (AOR: 2.34, 95% CI: 1.43-3.83, p<0.001).

Conclusion

This study reveals that early identification of IE in patients with septic shock is essential for improving outcomes. Key findings include the high diagnostic value of echocardiography and blood cultures in confirming IE, with prior cardiovascular disease emerging as a significant predictor of adverse outcomes. Embolic events and elevated inflammatory markers also played critical roles in predicting patient prognosis. A focused approach to early diagnosis, particularly through these key diagnostic tools, is crucial for timely intervention. Prioritizing these factors in clinical practice can help improve patient outcomes, especially in emergency and resource-limited settings.

## Introduction

A serious, sometimes fatal illness known as infectious endocarditis (IE) is typified by endocardial infection, frequently affecting the heart valves [1,2]. It is associated with high mortality and morbidity rates, with an estimated incidence of 3 to 10 cases per 100,000 person-years globally [3]. Despite advancements in diagnostic techniques and treatments, the in-hospital mortality rate for patients with IE remains significant, ranging from 15% to 25% [4]. IE presents significant management challenges, especially when complicated by septic coronary embolization, requiring multidisciplinary collaboration for diagnosis, treatment, and the management of complications like stroke and other embolic events, with no established guidelines for handling these cases [5]. The risk of adverse outcomes, including septic shock and embolic events, increases in these patients, underscoring the importance of early detection and intervention [6].

The causes of IE include bacteremia from sources such as intravenous drug use, prosthetic valve infections, and pre-existing valvular abnormalities [7]. Pathophysiologically, IE leads to endocardial infection and vegetation formation, which, when fragmented, can result in embolic complications such as stroke, splenic infarction, and limb ischemia. Septic shock in IE exacerbates hemodynamics through vasodilation, capillary leakage, and myocardial depression, while persistent inflammation and cardiac dysfunction may lead to refractory shock, further worsening the prognosis [8-10].

Septic shock, a severe and life-threatening form of sepsis, is characterized by profound metabolic and circulatory abnormalities, including persistent hypotension despite adequate fluid resuscitation and signs of organ dysfunction [11,12]. The overlap of symptoms, such as fever, hypotension, and organ dysfunction, makes distinguishing IE from other causes of septic shock challenging. Diagnostic tools such as echocardiography (both transthoracic and transesophageal) are critical for detecting vegetation, while biomarkers such as procalcitonin or C-reactive protein (CRP) may support early differentiation of IE. However, systemic inflammation often confounds clinical assessment, delaying timely diagnosis [13-15].

There is limited data on the clinical features and prognostic factors of IE in patients presenting with septic shock, which often leads to diagnostic delays and suboptimal outcomes. While the epidemiology and prognosis of IE and septic shock have been studied separately, little is known about how these conditions interact when occurring together in the emergency department. Combining the perspectives of IE and septic shock is critical, as it enables a comprehensive understanding of how systemic inflammation and cardiac infection interact, influencing outcomes. This approach may provide innovative insights into risk stratification and targeted management strategies for this high-risk population.

Understanding the clinical features and outcomes of IE in patients with septic shock can guide treatment decisions, improve risk stratification, and facilitate early diagnosis, ultimately enhancing patient outcomes in this high-risk population. Optimizing care pathways requires an understanding of the patterns and determinants of IE in this population, given the variability in presentation and resource limitations in emergency departments. The objective of this study is to explore the clinical characteristics, diagnostic findings, and treatment strategies in patients with infective endocarditis and septic shock, aiming to identify key predictors of adverse outcomes and contribute to evidence that may help refine clinical decision-making.

## Materials and methods

Study design and setting

This multicenter exploratory retrospective cohort study was conducted at three major tertiary care hospitals in Pakistan: Pakistan Institute of Medical Sciences (PIMS) Islamabad, Lady Reading Hospital Peshawar (LRH), and Lahore Medical Complex and The Heart Hospital (LMCH). The study spanned two years, from January 2022 to December 2023, and focused on patients presenting to the emergency departments with septic shock and subsequently diagnosed with IE.

Inclusion and exclusion criteria

Adults aged 18 years or older who presented with clinical signs of septic shock, as defined by the Sepsis-3 criteria, and were diagnosed with IE using the modified Duke criteria, were included in the study. Patients with other causes of shock (not related to sepsis), those with incomplete clinical or diagnostic information, and those who left against medical advice or were transferred to another facility before a diagnosis was confirmed were excluded.

Sample size

The Aga Khan University Hospital, Karachi, Pakistan (one of the largest tertiary care centers in the country) reported a 1.3% prevalence of sepsis among all hospital admissions [14]. The sample size was determined using the World Health Organization (WHO) sample size formula: n = \frac{Z^{2} \text{ x } P \textbf{ x } (1-p)}{d^{2}}, where n = required sample size, Z = standard normal deviation (typically 1.96 for a 95% confidence level), P = estimated prevalence of the condition in the population, 1 - P = complement of the prevalence (proportion without the condition), d = desired margin of error (precision level). The minimum required sample size was calculated to be 19.76 (approximately 20) patients per hospital. Given that three hospitals participated in the study, the adjusted minimum sample size was 60 patients. However, to enhance statistical power, ensure robust logistic regression analyses for identifying predictors of mortality and adverse outcomes, and improve external validity, the final sample size was expanded to 300 eligible participants, who were randomly selected across the three hospitals. Systematic patient selection ensured the representativeness of the broader population of septic shock patients with IE while minimizing selection bias.

Data collection

A systematic proforma was used to collect data prospectively, ensuring a structured and uniform approach across all participating sites. The proforma included specific fields for demographic information (e.g., age, sex, and relevant medical history), clinical presentation (e.g., presenting symptoms, signs at admission), diagnostic results (e.g., blood cultures, echocardiograms, inflammatory markers such as CRP and procalcitonin), treatment plans (e.g., antibiotic therapy, surgical interventions like valve replacement), and clinical outcomes (e.g., length of stay, in-hospital mortality, ICU admission).

Transthoracic echocardiography (TTE) and transesophageal echocardiography (TOE) were performed based on clinical indications. TTE was used as the first-line imaging modality for all patients due to its non-invasive nature and accessibility in critically ill patients with septic shock. If TTE provided inconclusive findings or if clinical suspicion of infective endocarditis (IE) remained high, TOE was performed for further evaluation. TOE was particularly considered in patients with prosthetic valves, intracardiac devices, previous history of IE, suspected paravalvular abscess, or suboptimal TTE image quality due to patient factors (e.g., obesity, mechanical ventilation). All echocardiographic assessments were interpreted by experienced cardiologists following the modified Duke criteria for the diagnosis of IE. The presence of vegetations, valve perforations, abscess formation, and regurgitant lesions were systematically recorded.

To ensure consistency in data collection, all data collectors underwent standardized training, including comprehensive instructions on how to complete the proforma. In addition, inter-rater reliability was assessed by periodically reviewing a random subset of patient data, with discrepancies discussed and addressed among the research team. This regular evaluation ensured high-quality, consistent data across all three centers.

Statistical analysis

The dependent variables in this study included in-hospital mortality, ICU admission, and adverse events such as embolic events and heart failure. Independent variables comprised demographic factors (age, gender), clinical features (hypotension, fever, dyspnea), diagnostic findings (echocardiographic positivity, blood culture positivity), and treatment strategies (antibiotics, valve surgery). Comorbidities, including hypertension, diabetes, and prior cardiovascular disease, as well as inflammatory markers, were included as covariates. To identify independent predictors of adverse outcomes, separate multivariate logistic regression analyses were performed for each outcome (in-hospital mortality, ICU admission, and adverse events). Data analysis was conducted using SPSS version 26, where categorical variables were presented as frequencies and percentages, and continuous variables were expressed as means ± standard deviation or medians with interquartile ranges. Chi-square tests were used for categorical variables, and Mann-Whitney U tests were applied for continuous variables depending on the normality of the data distribution. Missing data were handled using multiple imputation techniques to reduce bias and ensure robustness in the analyses. Multivariate logistic regression was employed to identify predictors of mortality, ICU admission, and adverse outcomes, with covariates selected based on their clinical relevance and statistical significance (p<0.10) from univariate analyses. The results were reported as adjusted odds ratios (AORs) with 95% confidence intervals (CIs), with a p-value of <0.05 considered statistically significant.

Ethical approval

Lady Reading Hospital Medical Teaching Institution Institutional Review Board (IRB) issued approval (Ref: No. 729/LRH/MTI. Dated: 20th Dec. 2021). Patients or their legal guardians provided written informed consent. Patient confidentiality was protected through the use of de-identified data with access restricted to authorized personnel; all analyses were conducted on anonymized datasets, and results were presented in aggregate to maintain privacy.

## Results

The baseline clinical and demographic characteristics of the 300 patients with infective endocarditis (IE) and septic shock are summarized in Table 1. The majority of patients were middle-aged, with a mean age of 55.20 ± 14.70 years. There was a predominance of males, with 190 patients (63.33%), compared to 110 females (36.67%). Hypertension was the most common comorbidity, present in 140 patients (46.67%), followed by diabetes mellitus in 100 patients (33.33%), chronic kidney disease (CKD) in 60 patients (20.00%), and prior cardiovascular disease (CVD) in 80 patients (26.67%). A smaller proportion of patients had a history of immunosuppression (25 patients, 8.33%) or previous IE (20 patients, 6.67%).

**Table 1:** Demographic and Clinical Characteristics Statistical analysis included chi-square (χ²) tests for categorical variables and independent t-tests for continuous variables. CVD included coronary artery disease (CAD), heart failure (HF), myocardial infarction (MI), atrial fibrillation (AF), valvular heart disease (VHD), prior cardiac surgery (CABG/valve replacement). Unknown sources of sepsis refer to cases where the primary infection site could not be determined despite clinical and diagnostic evaluation. Data was derived from patient records across three tertiary care hospitals, and inter-rater reliability was periodically assessed by reviewing a random subset of patient data. Discrepancies were discussed and addressed among the research team to ensure data consistency and accuracy. P-values of <0.05 were significant.

Category	Parameter	Frequency (%)	Mean ± SD	P-value	Statistical test
Age	Years	-	55.20 ± 14.70	0.147	t = 1.45
Gender	Male	190 (63.33)	-	0.292	χ² = 1.11
	Female	110 (36.67)	-	-	-
Comorbidities	Hypertension	140 (46.67)	-	0.355	χ² = 0.86
	Diabetes mellitus	100 (33.33)	-	0.71	χ² = 0.14
	Chronic kidney disease (CKD)	60 (20.00)	-	0.85	χ² = 0.04
	Prior cardiovascular disease (CVD)	80 (26.67)	-	0.204	χ² = 1.61
	Immunosuppression	25 (8.33)	-	0.27	χ² = 1.22
	History of infective endocarditis (IE)	20 (6.67)	-	-	-
Septic shock	Vasopressor use (Yes)	210 (70%)	-	0.012	χ² = 6.45
	SOFA Score ≥ 10	195 (65%)	-	0.009	χ² = 7.12

Regarding septic shock, 210 patients (70.00%) required vasopressor use, and 195 patients (65.00%) had a SOFA score of 10 or higher, indicating severe organ dysfunction. There were no significant differences in age, gender, or comorbidities such as hypertension, diabetes, CKD, prior CVD, and immunosuppression (p-values >0.05). The results were derived from patient records across three tertiary care hospitals, and inter-rater reliability was periodically assessed. Statistical tests revealed no significant differences between groups in these characteristics.

**Figure 1:**
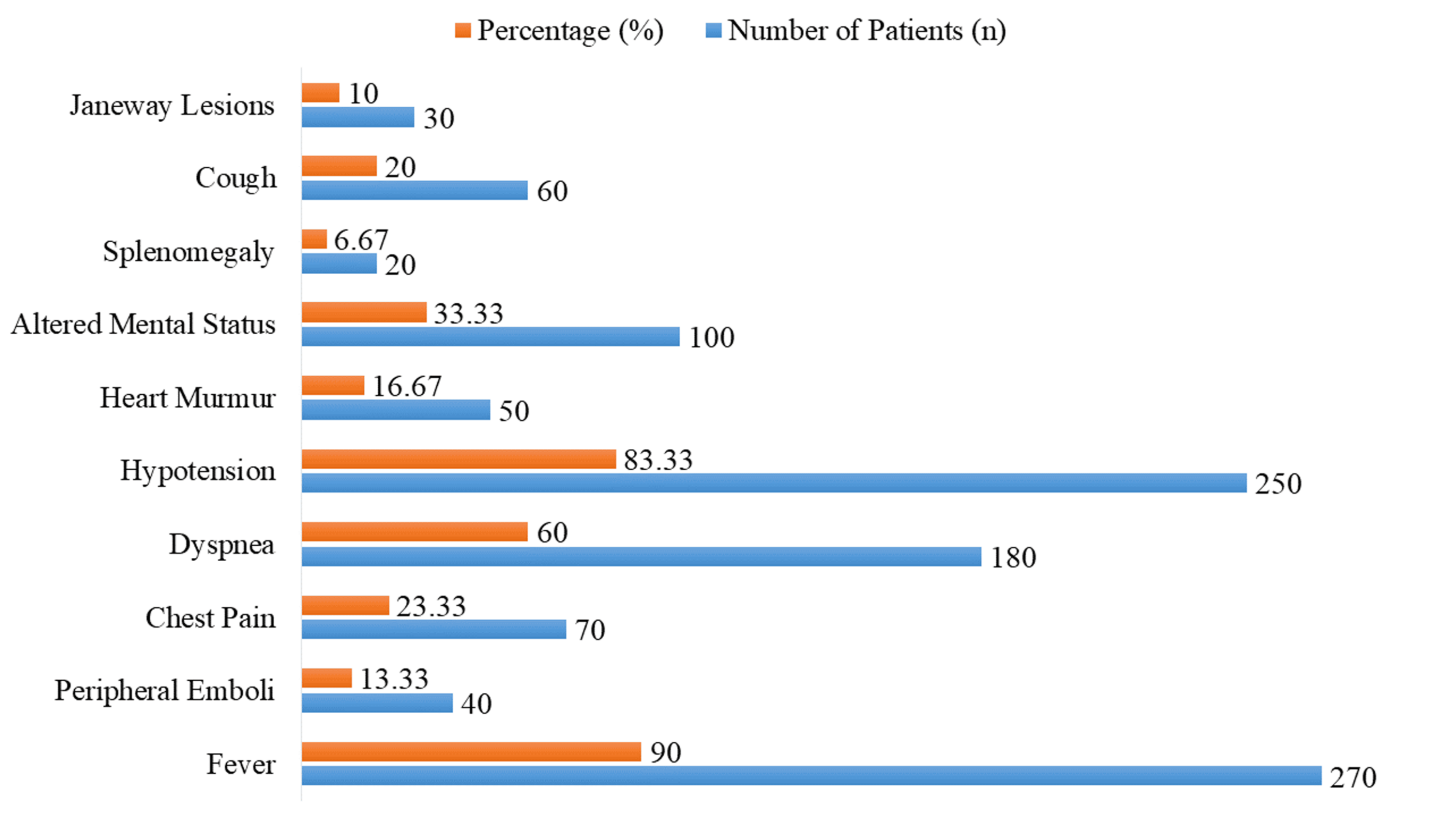
Clinical presentation of patients with infective endocarditis and septic shock

The clinical presentation is shown in Figure 1. Fever was the most common symptom, present in 270 patients (90.00%), followed by hypotension in 250 patients (83.33%) and dyspnea in 180 patients (60.00%). Altered mental status was noted in 100 patients (33.33%). Peripheral emboli were observed in 40 patients (13.33%), cardiac murmurs in 50 patients (16.67%), and Janeway lesions in 30 patients (10.00%). Additional symptoms included chest pain in 70 patients (23.33%), cough in 60 patients (20.00%), and splenomegaly in 20 patients (6.67%).

Table 2 shows the septic shock characteristics and microbiological findings in the 300 patients with IE. The source of sepsis could not be determined in 150 patients (50.00%). Among the known sources, respiratory infections were the most common (90 patients, 30.00%), followed by genitourinary infections (40 patients, 13.33%) and other sources (20 patients, 6.67%). The statistical analysis of microbial distribution across the sepsis sources did not show any significant differences (p-values >0.05). Regarding microbial findings,* Staphylococcus aureus *was the most frequently identified pathogen, found in 140 patients (50.00%), followed by *Streptococcus viridans *in 85 patients (30.36%), and *Enterococcus* spp. in 25 patients (8.93%). Other pathogens identified included *Escherichia coli* in 18 patients (6.43%) and *Klebsiella pneumoniae* in 12 patients (4.29%). The microbiological data showed that blood cultures were positive in 280 patients (93.33%) and that 278 patients (92.67%) had elevated inflammatory markers, reflecting the systemic involvement of the infection. The association between microbial distribution and sepsis source did not show significant statistical differences. These findings suggest that, despite the variability in sepsis sources, *Staphylococcus aureus* remained the dominant pathogen. 

**Table 2:** Septic shock and microbiological findings Data was derived from patient records across three tertiary care hospitals, and inter-rater reliability was periodically assessed by reviewing a random subset of patient data. Discrepancies were discussed and addressed among the research team to ensure data consistency and accuracy. P-values of <0.05 were significant.

Category	Parameter	Frequency (%)	P-value	Statistical test
Clinical features	Fever	270 (90.00)	0.802	χ² = 0.06
	Peripheral emboli	40 (13.33)	0.272	χ² = 1.21
	Chest pain	70 (23.33)	0.149	χ² = 2.08
	Dyspnea	180 (60.00)	0.828	χ² = 0.05
	Hypotension	250 (83.33)	0.147	χ² = 1.45
	Heart murmur	50 (16.67)	0.077	χ² = 3.12
	Altered mental status	100 (33.33)	0.357	χ² = 0.85
	Splenomegaly	20 (6.67)	0.837	χ² = 0.04
	Cough	60 (20.00)	0.252	χ² = 1.31
Valve involved	Mitral	35	0.015	χ² = 6.35
	Tricuspid	18	0.032	χ² = 4.78
	Aortic	70	0.0001	χ² = 12.89
	Pulmonary	5	0.74	χ² = 0.11
	Multiple valves	12	0.12	χ² = 2.39
Septic shock	Vasopressor use (yes)	210 (70%)	0.012	χ² = 6.45
	SOFA score ≥ 10	195 (65%)	0.009	χ² = 7.12
Source of sepsis	Unknown	150 (50.00)	-	-
	Respiratory	90 (30.00)	0.777	χ² = 0.08
	Genitourinary	40 (13.33)	0.792	χ² = 0.07
	Other	20 (6.67)	0.541	χ² = 0.37
Microbiological findings	Positive blood cultures	280 (93.33)	-	-
	Staphylococcus aureus	140 (50.00)	0.335	χ² = 4.56
	Streptococcus viridans	85 (30.36)		
	*Enterococcus *spp.	25 (8.93)		
	Escherichia coli	18 (6.43)		
	Klebsiella pneumoniae	12 (4.29)		

Table 3 summarizes the treatment strategies and outcomes for the 300 patients with IE and septic shock. The majority of patients received dual antibiotic therapy (290 patients, 96.67%) and supportive care, including IV fluids and vasopressors (286 patients, 95.33%). A smaller subset of patients, 50 patients (16.67%), underwent valve surgery. There were no significant differences in treatment strategies, including antibiotics (p=0.92), valve surgery (p=0.51), or supportive care (p=0.88). Regarding patient outcomes, 51 patients (17.00%) died in the hospital, while 99 patients (33.00%) experienced embolic events. Approximately half of the patients (150 patients, 50.00%) required ICU admission due to critical illness, comorbidities, and complications. The mean hospital stay was approximately 18.5 days, with prolonged hospitalization linked to increased complications. There were no significant differences in in-hospital mortality (p=0.29), embolic events (p=0.80), or ICU admission (p=0.56) across treatment groups. Despite these findings, the data reveal trends suggesting that longer hospital stays and a higher incidence of embolic events are associated with worse outcomes. These results highlight the need for early intervention and close monitoring, especially in patients with prolonged stays and severe comorbidities. 

**Table 3:** Diagnostic findings, treatment strategies, and length of stay Data was derived from patient records across three tertiary care hospitals, and inter-rater reliability was periodically assessed by reviewing a random subset of patient data. Discrepancies were discussed and addressed among the research team to ensure data consistency and accuracy. P-values of <0.05 were significant. CRP - C-reactive protein; IE - infective endocarditis; ESR - erythrocyte sedimentation rate

Category	Parameter	Frequency (%)	Mean ± SD	P-value	Statistical test
Diagnostic findings	Echocardiographic confirmation of IE	289 (96.33)	-	-	-
	Blood cultures positive	278 (92.67)	-	-	-
	Elevated inflammatory markers (CRP, ESR)	260 (86.67)	-	-	-
Treatment strategies	Antibiotics (dual therapy)	290 (96.67)	-	0.928	χ² = 0.01
	Valve surgery	50 (16.67)	-	0.514	χ² = 0.42
	Supportive care (IV fluids, vasopressors)	286 (95.33)	-	0.883	χ² = 0.02
Length of stay	Length of stay (days)	-	18.53 ± 5.01	-	-

In the septic shock management data (Table 4), 70% (n=210) of patients required vasopressor use (norepinephrine), and 40% (n=120) needed mechanical ventilation. Lactate levels averaged 3.8 ± 1.5 mmol/L, while the mean arterial pressure (MAP) was 62.5 ± 10.2 mmHg. Regarding outcomes, 17% (n=51) of patients experienced in-hospital mortality, with 33% (n=99) suffering from embolic events. Additionally, 50% (n=150) of patients were admitted to the ICU. Statistical tests showed no significant p-values for mortality, complications, and ICU admission (p=0.295, 0.803, and 0.564, respectively).

**Table 4:** Septic shock management and outcomes

Category	Parameter	Frequency (%)	Mean ± SD	P-value	Statistical test
Septic shock management	Vasopressor use (norepinephrine)	210 (70%)	-	-	-
	Mechanical ventilation required	120 (40%)	-	-	-
	Lactate levels (mmol/L)	-	3.8 ± 1.5	-	-
	Mean arterial pressure (MAP, mmHg)	-	62.5 ± 10.2	-	-
Outcomes	In-hospital mortality	51 (17.00%)	-	0.295	χ² = 1.10
	Complications (embolic events)	99 (33.00%)	-	0.803	χ² = 0.06
	ICU admission	150 (50.00%)	-	0.564	χ² = 0.33

Effect sizes, measured using Phi (φ) and Cramer's V, indicated small relationships across most variables, aligning with the non-significant p-values. Despite the lack of statistical significance, the data reveal notable trends, including the predominance of respiratory sources of sepsis (30%) and low rates of peripheral embolic events (13.33%) and splenomegaly (6.67%). These trends, while not definitive, provide insight into the clinical landscape of IE and septic shock. Age, hypotension, and echocardiography positivity are key predictors of adverse outcomes such as mortality, prolonged hospital stay, and complications like embolic events and organ dysfunction. Each year of age increases the risk of adverse outcomes by 5% (AOR: 1.05, 95% CI: 1.02-1.08, p=0.001), and a 10-year age difference can raise this risk by 50%. Hypotension (AOR: 2.36, 95% CI: 1.43-3.92, p<0.001) and echocardiography positivity (AOR: 2.43, 95% CI: 1.36-4.34, p<0.001) emphasize the need for early, aggressive management in these patients, as shown on Table 5).

**Table 5:** Multivariate logistic regression analysis for predictors of mortality and adverse outcomes in patients with infective endocarditis and septic shock

Predictor	Adjusted odds ratio (AOR)	95% Confidence interval (CI)	P-value
Age (per year increase)	1.05	1.02 - 1.08	0.001
Gender (male vs. female)	1.12	0.70 - 1.78	0.619
Hypertension	1.30	0.83 - 2.04	0.235
Diabetesmellitus	0.92	0.57 - 1.49	0.724
Chronic kidney disease	1.44	0.76 - 2.73	0.259
Prior cardiovascular disease	2.14	1.12 - 4.08	0.021
Immunosuppression	1.52	0.71 - 3.23	0.276
Source of sepsis (respiratory vs. others)	0.87	0.54 - 1.41	0.590
Echocardiography positive	2.43	1.36 - 4.34	<0.001
Hypotension	2.36	1.43 - 3.92	<0.001
Heart murmur	1.29	0.74 - 2.26	0.371
Blood cultures positive	2.50	1.43 - 4.34	<0.001
Embolic events	3.10	1.86 - 5.14	<0.001
Length of stay (per day increase)	1.11	1.06 - 1.16	<0.001
Elevated inflammatory markers	2.34	1.43 - 3.83	<0.001

The wide confidence intervals for predictors like prior cardiovascular disease (AOR: 2.14, 95% CI: 1.12-4.08, p=0.021) and embolic events (AOR: 3.10, 95% CI: 1.86-5.14, p<0.001) suggest variability in their effects or potential heterogeneity in the study population. This variability could stem from differences in baseline health status, comorbidity profiles, or treatment delays. Non-significant predictors, such as gender (AOR: 1.12, 95% CI: 0.70-1.78, p=0.619) and immunosuppression (AOR: 1.52, 95% CI: 0.71-3.23, p=0.276), may challenge prior assumptions or reflect cohort-specific dynamics. For instance, male predominance in this study (63.33%) might dilute the statistical significance of gender as a risk factor. 

The significance of the length of stay (AOR: 1.11 per day, 95% CI: 1.06-1.16, p<0.001) highlights the cumulative burden on healthcare resources and patient outcomes. A 10-day increase corresponds to an approximately 11-fold rise in adverse outcomes. This underscores the importance of expedited care pathways and timely interventions. Interactions between age and comorbidities, such as chronic kidney disease or hypertension, merit further exploration, as they may amplify the risks associated with individual predictors. These findings translate into actionable clinical strategies. Older patients or those presenting with hypotension should be triaged for more intensive monitoring and management. Routine echocardiography and expedited blood cultures are indispensable for early detection and intervention in IE cases. The heightened attention to embolic events is warranted, given their strong association with adverse outcomes.

## Discussion

Building upon the aforementioned background, our study aims to address a gap in the existing literature by specifically focusing on the clinical features and outcomes of IE in patients presenting with septic shock. While both conditions are associated with significant morbidity and mortality, there remains a lack of comprehensive data on their concurrent occurrence and the impact of this combination on patient prognosis. Our findings emphasize that middle-aged male patients with comorbidities, including hypertension, diabetes mellitus, and chronic kidney disease, are most commonly affected. Respiratory infections emerged as the leading source of sepsis, and Staphylococcus aureus was the most frequently identified pathogen. A substantial proportion of the cohort exhibited echocardiographic findings consistent with IE, which highlights the critical importance of early imaging in diagnosing and managing these patients. Most of the patients received dual antibiotic therapy and supportive care, contributing to better outcomes. Our analysis reveals that key factors such as age, hypotension, and echocardiographic positivity were strong predictors of adverse outcomes, including higher mortality, prolonged hospital stays, and complications such as embolic events and organ dysfunction. Through this detailed analysis, we aim to identify essential predictors for these adverse outcomes, underscoring the need for timely, targeted interventions. By integrating insights from both IE and septic shock, this study provides valuable information on how these conditions interact and emphasizes the need for early risk stratification and optimized treatment strategies to improve patient outcomes in this high-risk population.

Our demographic findings align with previous research indicating a higher prevalence of IE in older male populations, though the average age of 55.20 years in our study represents a younger subset compared to some studies on "older" patients [15]. The male predominance (63.33%) warrants further exploration regarding its implications for diagnosis, treatment, and outcomes. The prevalence of comorbidities such as chronic renal disease, diabetes, and hypertension, consistent with prior research, was explored in relation to their impact on the outcomes of both IE and septic shock [13,16]. Interestingly, the absence of other comorbidities, such as intravenous drug use or valve-related conditions, may reflect the unique demographics of our study population. The high proportion of unknown sepsis sources (50.00%) further highlights challenges in the diagnostic workup and the timing of presentation, a concern also raised in other studies [15-17]. Embolic events (33.00%) and respiratory sources (30.00%) were prominent, while the mortality rate (17.00%) and lower rates of genitourinary sepsis (13.33%) were observed to differ from those in prior studies, potentially reflecting regional infection patterns or variations in diagnostic protocols [18]. The relatively low rates of peripheral emboli (13.33%) and cardiac murmurs (16.67%) might also be due to delayed presentation or limitations in imaging methods [14]. Additional symptoms, such as altered mental status and splenomegaly, were highlighted to enhance the clinical profile of this patient group [19].

Echocardiography and blood cultures were essential in diagnosing IE in our cohort, with sensitivity rates of 96.33% and 92.67%, respectively, underscoring their diagnostic value in this setting [20]. The prognostic significance of inflammatory markers, such as C-reactive protein (CRP) and erythrocyte sedimentation rate (ESR), was also discussed, emphasizing their correlation with disease severity and timing of intervention [20]. Mortality rates, embolic events, and the mean hospital length of stay (18.53 ± 5.01 days) in our cohort align with similar studies, with outcomes influenced by factors like the timing of intervention and disease severity at presentation [21,22]. The multivariate analysis identified age, hypotension, and echocardiographic positivity as strong predictors of adverse outcomes, including mortality, prolonged hospitalization, and embolic events. Specifically, each additional year of age was associated with a 5% increase in the risk of adverse outcomes (AOR: 1.05, 95% CI: 1.02-1.08, p=0.001), while hypotension (AOR: 2.36, 95% CI: 1.43-3.92, p<0.001) and echocardiographic positivity (AOR: 2.43, 95% CI: 1.36-4.34, p<0.001) emerged as key predictors, emphasizing the need for aggressive hemodynamic stabilization and early imaging-based surveillance.

Embolic events (AOR: 3.10, 95% CI: 1.86-5.14, p<0.001) were identified as a significant determinant of poor prognosis, highlighting the importance of vigilant monitoring and early intervention in this patient population. Although small effect sizes were measured by Phi (φ) and Cramer's V, our findings suggest notable trends, such as the predominance of respiratory infections as the primary source of sepsis (30%) and lower rates of peripheral embolic events (13.33%) and splenomegaly (6.67%). The length of hospital stay was also identified as a key factor, with each additional day associated with an 11-fold increase in adverse events (AOR: 1.11 per day, 95% CI: 1.06-1.16, p<0.001). Additionally, prior cardiovascular disease (AOR: 2.14, 95% CI: 1.12-4.08, p=0.021) showed variability in predictive strength, warranting further investigation. Non-significant predictors, such as gender (AOR: 1.12, 95% CI: 0.70-1.78, p=0.619) and immunosuppression (AOR: 1.52, 95% CI: 0.71-3.23, p=0.276), indicate cohort-specific dynamics that may dilute statistical significance. These findings have important clinical implications, emphasizing the need for tailored risk stratification, intensive monitoring of high-risk patients, and expedited diagnostic pathways to improve patient outcomes. Further studies should explore the integration of embolic risk stratification tools and examine the long-term impact of these predictors on patient outcomes.

Lastly, we acknowledge that non-significant predictors, such as diabetes and chronic kidney disease, warrant further exploration to determine their potential role in patient outcomes, as they may align with existing literature [23]. This study identifies critical predictors of adverse outcomes in patients with infective endocarditis (IE) and septic shock, including age, embolic events, and inflammatory markers. These findings have significant clinical relevance, as they can guide early intervention strategies and resource allocation. Older patients, particularly those with embolic events, may benefit from more aggressive monitoring and timely surgical intervention. Additionally, inflammatory markers like CRP and ESR can aid in risk stratification, helping to tailor treatment plans. Recognizing these predictors highlights the importance of diagnostic consistency and early intervention to improve patient outcomes.

Recent studies highlighted the importance of early echocardiography and surgical consultations to reduce adverse outcomes in similar patient populations [24,25]. Our study's findings support the need for protocol updates in emergency departments and tertiary care centers to optimize care. Although limitations such as observational biases, unmeasured variables like socioeconomic status, and the absence of long-term follow-up must be acknowledged, future research should explore the long-term outcomes of patients with septic shock and IE in different clinical settings. The multicenter design of our study strengthens the external validity of the findings, while the use of modified Duke criteria ensures diagnostic reliability. Given the variability in predictors such as prior cardiovascular disease, further investigation is necessary to better understand their impact on patient outcomes. Despite these limitations, the findings offer valuable insights for improving hospital protocols and guiding early intervention in high-risk patients.

Strengths and limitations

This study's strengths include its two-year multicenter design, which enhances the generalizability of our findings across diverse clinical settings. The use of modified Duke criteria ensures diagnostic accuracy and reliability, while standardized data collection methods strengthen the validity of the results. A sample size of 300 patients provides sufficient statistical power to identify meaningful associations between patient characteristics and outcomes. Despite these strengths, there are limitations. As an observational study, we cannot draw definitive causal conclusions, and unmeasured confounders, such as socioeconomic status, healthcare access, and institutional care protocols, may have influenced our results. Selection bias is another limitation, as we included only patients from tertiary care centers, which may affect the generalizability of our findings to under-resourced settings. The retrospective nature of the study introduces the potential for misclassification bias, but we employed measures to reduce this risk. Additionally, the limited number of patients who underwent surgery may have impacted the statistical power to detect differences in outcomes between the surgically and medically managed groups. Finally, while the two-year duration is relatively long, it does not capture long-term morbidity or sequelae, which will be an important focus of future studies. Further research incorporating extended follow-up periods and expanding to under-resourced populations is needed to fully understand the long-term outcomes of this patient group.

## Conclusions

This multicenter observational study provides comprehensive insights into the incidence, clinical features, and outcomes of IE in patients with septic shock. By including diverse geographic and demographic settings, the study enhances the generalizability of its findings across various clinical environments. The cohort predominantly comprised middle-aged males with significant comorbidities such as hypertension, diabetes mellitus, and chronic kidney disease. Fever, hypotension, and dyspnea were the most prevalent clinical symptoms. Importantly, echocardiography and blood cultures were identified as the most reliable diagnostic tools, with IE confirmed by echocardiographic findings, demonstrating a strong association between clinical diagnosis and imaging results. The modified Duke criteria, integrating clinical, microbiological, and echocardiographic parameters, further enhanced diagnostic accuracy, making it especially relevant in this high-risk patient population.

The study highlights key predictors of adverse outcomes, including advanced age, prior cardiovascular disease, echocardiographic positivity, blood culture positivity, and embolic events. These findings emphasize the importance of early diagnosis and targeted management strategies to optimize patient outcomes in emergency department settings. With a large sample size and standardized data collection methods, this study offers robust evidence for the critical role of early intervention and continuous monitoring in patients with IE and septic shock. Further research is needed to explore the impact of socioeconomic disparities and healthcare access, particularly in under-resourced settings, to improve the representativeness and generalizability of these findings.
